# The success and effectiveness of miniscrew-assisted rapid palatal expansion are age- and sex-dependent

**DOI:** 10.1007/s00784-021-04281-0

**Published:** 2021-11-25

**Authors:** Ji Yoon Jeon, Sung-Hwan Choi, Chooryung Judi Chung, Kee-Joon Lee

**Affiliations:** 1 Department of Orthodontics, Institute of Craniofacial Deformity, Yonsei University College of Dentistry, 50-1 Yonsei-ro, Seodaemun-gu, Seoul, 03722 Korea; 2 Department of Orthodontics, Gangnam Severance Hospital, 211, Eonju-ro, Gangnam-gu, Seoul, Korea; 3 Institute of Craniofacial Deformity, Yonsei University College of Dentistry, Seoul, 06273 Korea

**Keywords:** Palatal expansion technique, Orthodontic appliances, Adult, Cranial sutures

## Abstract

**Objectives:**

This study aimed to assess the success rate and the amount of suture separation after the miniscrew-assisted rapid palatal expansion (MARPE) procedure in relation to the chronological age and sex of the patients.

**Materials and methods:**

The periapical radiographs of 215 subjects (95 male; 120 female; range, 6–60 years) who had undergone MARPE treatment were retrospectively analyzed. The success of suture separation was determined and, in suture-separated subjects, the amount of suture separation was evaluated by suture separation ratio calculated from the periapical radiograph obtained after active expansion. Association tests were performed using linear-by-linear association, the Jonckheere-Terpstra test, Fisher’s exact test, and the Mann–Whitney *U* test, and linear regression models were also developed.

**Results:**

The success rate of suture separation was 61.05% in male, 94.17% in female, and 79.53% in both sexes. There was a statistically significant association between older age and suture nonseparation in male (*p* < 0.001), but not in female (*p* = 0.221). In suture-separated subjects, there was a statistically significant trend toward a low amount of suture separation with older age subgroups in both sexes (*p* < 0.001); however, there was no statistically significant difference in the amount of suture separation between male and female in all age subgroups.

**Conclusions:**

Older patients treated with MARPE, particularly in male, may have a reduced likelihood of both success in suture separation and sufficient basal bone expansion.

**Clinical relevance:**

This study demonstrates that clinicians should consider that the success rate of MARPE and the amount of suture separation may depend on chronological age and sex.

**Supplementary Information:**

The online version contains supplementary material available at 10.1007/s00784-021-04281-0.

## Introduction

Maxillary transverse deficiency can manifest as unilateral or bilateral posterior crossbites, as well as crowding or protrusion in the maxillary arch. Rapid palatal expanders are used for the resolution of maxillary transverse deficiency [1, 2], mainly in children and adolescents under 15 years of age, possibly because of the gradual increase in resistance from the interdigitation of the midpalatal suture with age [3, 4]. Radiographic confirmation of the separation of the midpalatal suture along with the midline diastema is a strong indicator of successful orthopedic expansion. Alternative surgically assisted palatal expansion has been indicated in postpubertal and mature patients [5]. Recently, the combined use of bone-borne miniscrews connected to hyrax expanders has resulted in successful non-surgical orthopedic expansion in adults of various ages, including those in their 20 s and 30 s [6–9]. The rationale for the incorporation of miniscrews includes a possible increase in the success rate and/or safety during and after expansion to protect the buccal plate of the anchor teeth against the pressure from the hyrax expander [10]. However, despite the promising outcomes in adults, the success rate and the quality of expansion related to age or sex remain unclear.

As regards the success rate of miniscrew-assisted rapid palatal expansion (MARPE), previous studies mainly focused on young adults in their late teens or early 20 s, but the number of participants was insufficient to identify a reliable pattern [6, 8, 9, 11]. According to the meta-analysis of Kapetanović et al. [6], the success rate of MARPE varied from 80.65 to 100% including only limited numbers of older patients within the sample size of 8 to 69. Choi et al. [9] reported a success rate of 87% in 69 young adults, and Oliveira et al. [8] reported a success rate of 71.4% in 28 adults. Regarding sex, Oliveira et al. [8] demonstrated that the success of MARPE was not correlated with sex; however, in contrast, Choi et al. [9] reported that the incidence of suture nonseparation was higher in male. Thus, the literature is somewhat contrasting, which might be a result of the random errors due to the small sample size with limited distribution of a wide range of chronological age or sex [12]. Additionally, studies on the amount of suture separation in suture-separated patients using the suture separation ratio or its correlation with chronological age and sex are still limited.

Previous studies indicated that chronological age should be considered as an important factor of success in maxillary expansion because the maturation and articulation of the midpalatal and other circummaxillary sutures increase with age [3, 4]. However, according to Perrson et al. [4], Knaup et al. [13], and Melsen [14], the maturation of the midpalatal suture starts from the posterior area with increased individual variation in interdigitation, obliteration, and ossification values, thereby the time point of complete ossification may not be proportional to chronological age. Practically, adult patients aged 27, 32 [4], 54 [13], and even 71 [15] years have been reported to have no signs of fusion, and Capelozza et al. [16] and Handelmann et al. [17] demonstrated success using conventional rapid palatal expanders in adults. Moreover, Wehrbein et al. [18] stated that the patency of the midpalatal suture should not be considered as a sole factor for the success of orthopedic expansion, implying the role of other sutures in successful suture separation.

Due to the variability in the literature, ensuring expansion at a specific age or in a particular sex remains a challenge among clinicians. Although the assessment of midpalatal suture maturation has been suggested as a possible determinant for successful orthopedic expansion [19, 20], it is reportedly impossible to determine obliteration using regular radiological equipment [18] because histological analysis cannot be performed in living patients, and it is barely possible to assess from a microscopic perspective even with computed tomography (CT) images. Meanwhile, the prediction of the success or the possible amount of expansion by determining the approximate trend of nonsurgical maxillary expansion according to chronological age and sex through general macroscopic studies would be of practical support in strategic treatment planning. Thus, this study aimed to investigate the success rate and the amount of suture separation after the MARPE procedure in relation to patients’ chronological age and sex.

## Materials and methods

### Subjects

This retrospective study included 227 patients with transverse maxillary deficiency who underwent MARPE treatment between January 2004 and May 2020 at the Department of Orthodontics, Yonsei University Dental Hospital, Seoul, Korea. The study protocol was approved by the Institutional Review Board of Yonsei Dental Hospital (IRB No. 2–2020-0031). Ethical approval was waived in view of the retrospective nature of the study and all the procedures being performed were part of the routine care. The investigation was performed in accordance with the Declaration of Helsinki (2013).

The inclusion criteria were as follows: maxilla-mandibular intermolar width difference less than the normal value, availability of a complete series of identifiable periapical radiographs of the maxillary central incisor and jackscrew of the appliance, no prior history of orthodontic treatment and/or orthognathic surgery, no severe dentofacial anomalies such as a cleft lip or palate, good oral hygiene, and healthy periodontal tissues. Regardless of the chronological age and sex, under the presumption that the maxilla-mandibular intermolar width difference must be constant for normal occlusion, 8.43 ± 2.22 mm was used as the reference for normal occlusion based on a previous study on Koreans [21].

**Fig. 1:**
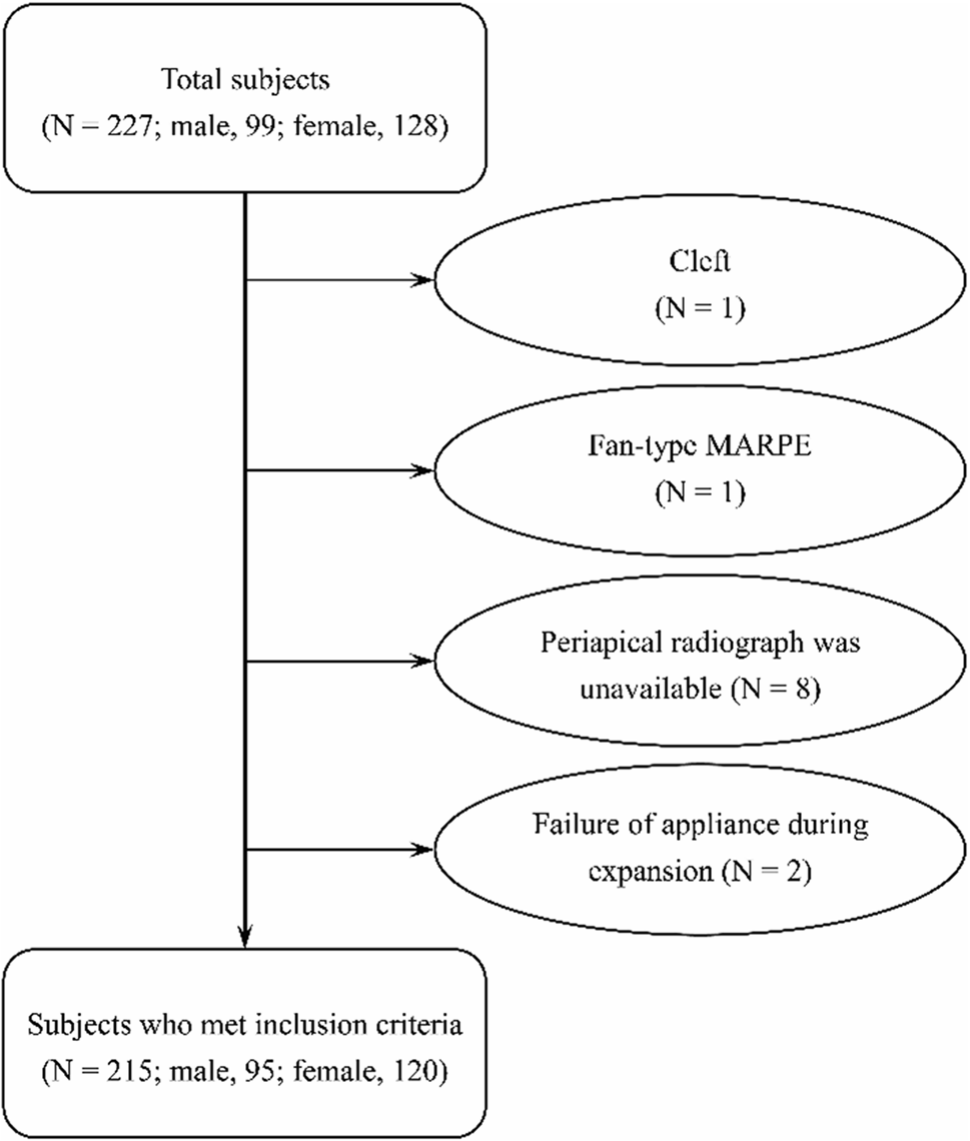
Flow chart of study enrollment

Of the 227 potentially eligible patients, 215 (95 male and 120 female) met the inclusion criteria and were selected for analysis regardless of age, sex, and success of MARPE (Fig. 1). The mean age at the start of expansion was 20.0 ± 7.3 years (range, 6–60 years; median, 19.0). The overall age and sex distribution of subjects is shown in Table 1.

**Table 1:** Participant characteristics

	Total (*N* = 215)	Male (*N* = 95)	Female (*N* = 120)
Age at treatment (year)	20.0 ± 7.3^*a*^ (range, 6–60; median, 19.0)	20.5 ± 5.4^*a*^ (range, 8–38; median, 21.0)	19.6 ± 8.5^*a*^ (range, 6–60; median, 18.5)
Age group (year)			
≤15	56	17	39
16–20	71	27	44
21–25	55	38	17
26–30	19	10	9
31–35	6	2	4
36–40	4	1	3
> 40	4	0	4

^*a*^Values are expressed as means ± standard deviations

### Appliance and clinical protocol

**Fig. 2:**
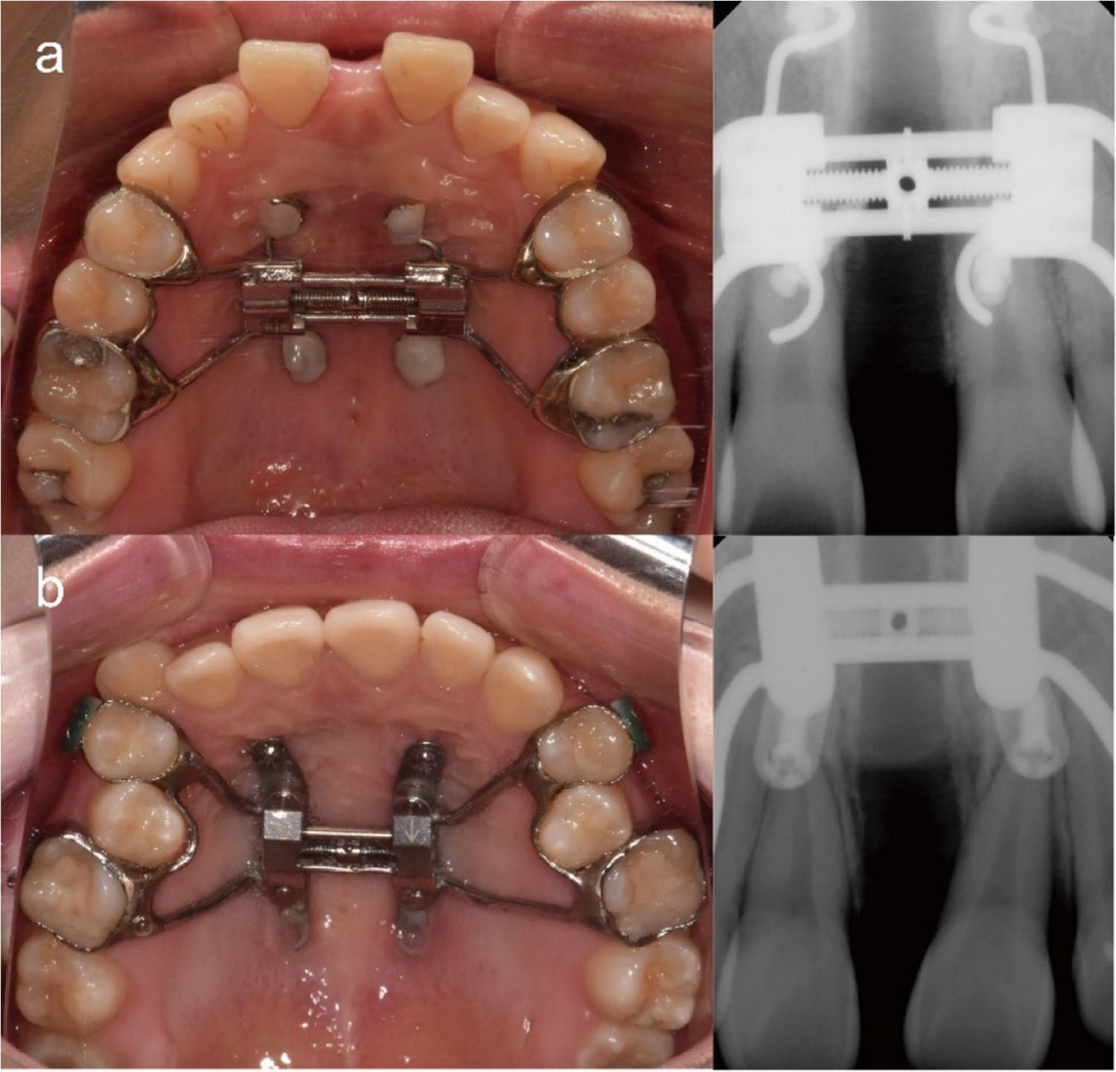
Miniscrew-assisted rapid palatal expansion appliances and periapical radiographs after expansion. **a** Semi-rigid type. **b** Rigid type

Depending on time, two types of semi-rigid (four rigid stainless steel wire connectors with helical hooks) and rigid (two metal plates with screw holes) type MARPE appliances were used according to the development of the appliance manufacturing method. In both types, connectors for miniscrews were soldered to the base of the hyrax screw (Biomaterials Korea, Kee’s Bone Expander, Seoul, Korea) to be placed on the anterior and posterior sides of the jackscrew. The maxillary first premolars and first molars were used as anchor teeth; however, the first or second deciduous molars were selected if the first premolar had not erupted. Four miniscrews (diameter, 1.8 mm; length, 9.0 mm for the anterior side of jackscrew; length, 7.0 mm for the posterior side of jackscrew; self-drilled type, BMK, Ortholution, Seoul, Korea) were inserted perpendicular to the center of the helical hooks (diameter, 4.0 mm) or metal plates under local infiltration anesthesia after the MARPE appliance was placed and cemented on the maxillary dentition (Fig. 2). While using the semi-rigid type MARPE, the heads of the miniscrews were covered with light-cured resin (Transbond, 3 M Unitek, St Paul, MN, USA) to connect the miniscrews with the helical hooks, minimize irritation, and improve postinsertion stability.

**Fig. 3:**
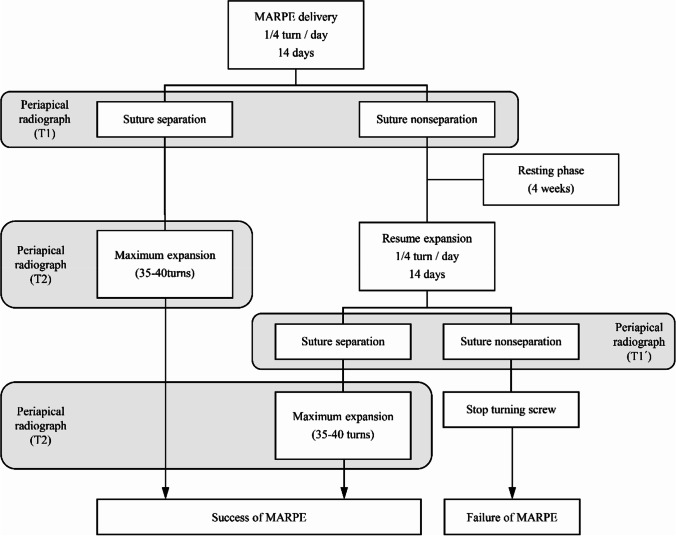
Allocation of success and failure in suture separation

The MARPE device was activated by one turn/day with one-quarter of a turn (0.2 mm/turn). During MARPE treatment, periapical radiographs of the maxillary central incisor were acquired, including the jackscrew of the MARPE device via the paralleling technique to avoid distortion of the image before treatment (T0), after 14 days of turning the screw (T1), and after active expansion (T2) in suture-separated subjects. If the suture did not separate, a periapical radiograph was not acquired at T2 (Fig. 3).

After 14 days of turning the screw, midpalatal suture separation was assessed through the periapical radiograph acquired at T1 by evaluating whether the midpalatal suture had split and the inter-sutural gap had increased compared to the periapical radiograph acquired at T0. In the case of suture nonseparation, the subject underwent a 4-week resting phase, and expansion resumed thereafter. Reevaluation of suture separation was performed after an additional 14 days of turning the screw, and expansion was discontinued if the suture was nonseparated again (Fig. 3). Active expansion was performed until the palatal cusp of either of the maxillary anchor teeth came into contact with the buccal cusp of the corresponding mandibular teeth and was followed by a 3–6 month retention period to allow bone formation in the separated midpalatal suture and to prevent skeletal relapse.

### Measurements

**Fig. 4:**
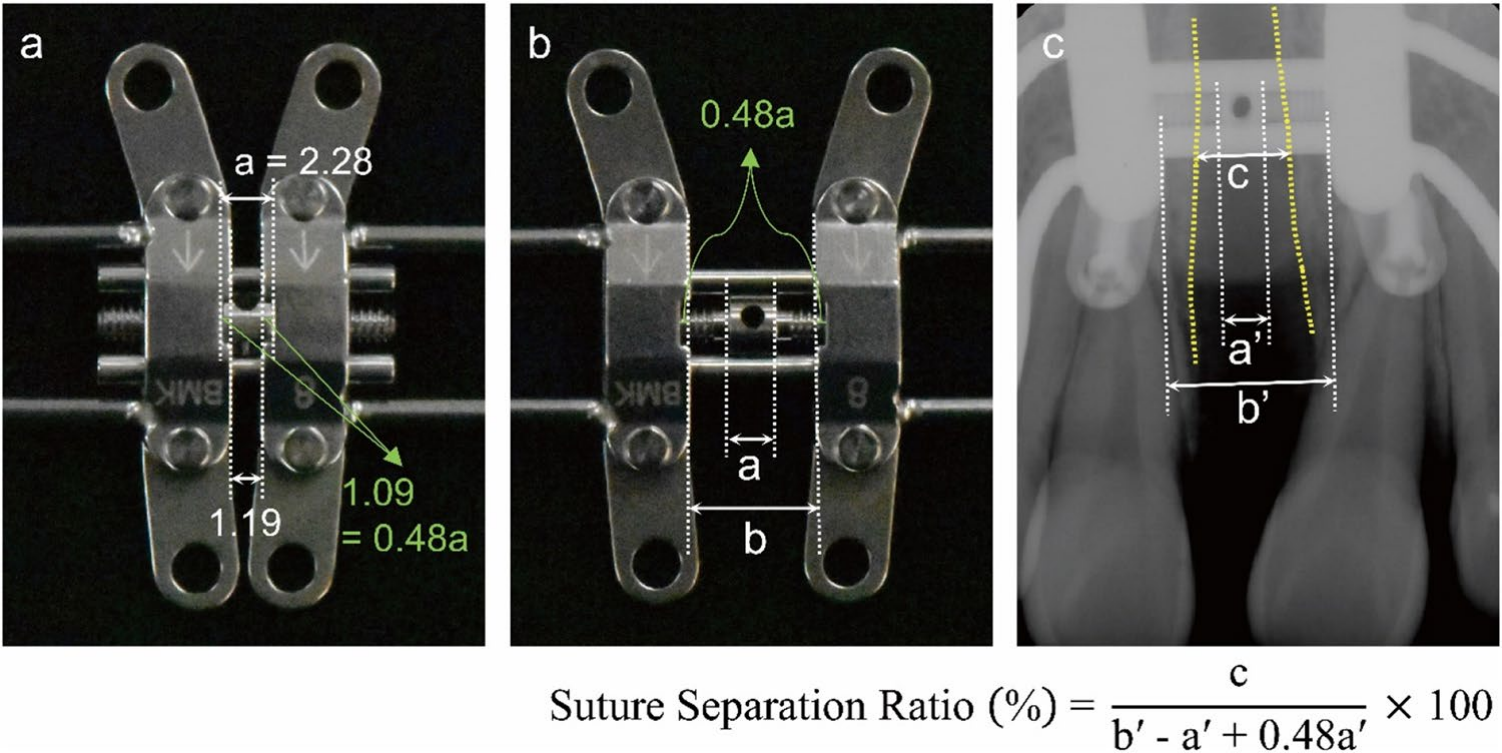
Measurements of each variable (mm) and calculation of the suture separation ratio (%). **a** Calculation of the initial step (0.48a) within the jackscrew before expansion in unit of slot width. **b** Jackscrew after expansion. a: Slot width. b: Jackscrew expansion width. **c** Measurement of variables in the periapical radiograph. a’: Slot width in the periapical radiograph, b’: Jackscrew expansion width in the periapical radiograph. c: Midpalatal suture expansion width

In suture-separated subjects, three parameters of jackscrew expansion width, slot width, and midpalatal suture expansion width were measured in the periapical radiograph of T2 to evaluate suture expansion amount using ZeTTA PACS Viewer (Taeyoung Soft Company, Korea) by one researcher (Table 2, Fig. 4). As a standardized ratio value of the midpalatal suture expansion amount compared with the pure jackscrew expansion amount under relatively similar condition, the suture separation ratio was calculated considering the magnification ratio. Meanwhile, for subjects who were treated with rigid type of MARPE appliance, the existing initial step within the jackscrew needed calibration, and a modified equation with a calculated value of 0.48×slot width, was applied.

**Table 2:** Definitions of the parameters measured in the study

Measurement	Definition
Jackscrew expansion width (mm)	The shortest distance between the two points located at the anterior border of expanded jackscrew after maximum expansion on periapical radiograph (refer to Fig. 4c, measurement b’)
Slot width (mm)	Slot width of jackscrew at the most anterior side on periapical radiograph (refer to Fig. 4c, measurement a’)
Midpalatal suture expansion width (mm)	The shortest distance between the two points located at both cortical bone layers of separated midpalatal suture to be on the same line as jackscrew expansion width on periapical radiograph (refer to Fig. 4c, measurement c)
Suture separation ratio (%)	$\frac{\text{Midpalatal suture expansion width}}{\mathrm{Jackscrew expansion width}-\mathrm{Slot width}}\times 100 (\%)$ (refer to Fig. 4)

### Reliability

All evaluations and measurements were performed by a single trained and calibrated investigator. Reproducibility was determined by comparing repeated measurements for each variable, with a 2-week interval between measurements. The intra-class correlation coefficient was greater than 0.95 for all variables measured in this study.

### Statistical analysis

All statistical analyses were performed using IBM SPSS software for Windows (version 20.0; SPSS Inc., Chicago, IL, USA). A *p* value less than 0.05 was considered statistically significant.

The associations between age subgroup and either success of suture separation or suture separation ratio in each sex were evaluated using linear-by-linear association and the Jonckheere-Terpstra test. In each age subgroup, associations between sex and the success of suture separation were evaluated using Fisher’s exact test, and the Mann–Whitney *U* test was applied to compare the suture separation ratio between sexes.

Correlations between the suture separation ratio and age as numerical data for each sex were evaluated using the Pearson correlation coefficient. The Shapiro–Wilk test was used to determine the normality of the data distributions. With regard to the strengths of the correlations, *r* > 0.90 indicated a very high correlation, 0.70 < *r* < 0.90 indicated a high correlation, 0.50 < *r* < 0.70 indicated a moderate correlation, 0.30 < *r* < 0.50 indicated a low correlation, and *r* < 0.30 indicated a negligible correlation [22]. Finally, a linear regression model was developed.

## Results

### Success rate of suture separation

Table 3 shows the descriptive statistics and associations among the age subgroups, sex, and the success of suture separation.

**Table 3:** Descriptive statistics and associations among age subgroup, sex, and the success of suture separation

Age subgroup		Success of suture separation			Success rate (%)		
		Sep	Nonsep	Subtotal	Each sex	*p* (Fisher’s exact test)	Each age subgroup
6-15Y (*N* = 56)	M	16	1	17	94.12	0.501	96.43
	F	38	1	39	97.44		
16-20Y (*N* = 71)	M	22	5	27	81.48	0.248	88.73
	F	41	3	44	93.18		
21-25Y (*N* = 55)	M	16	22	38	42.11	< 0.0001****	58.18
	F	16	1	17	94.12		
26-30Y (*N* = 19)	M	4	6	10	40.00	0.011*	68.42
	F	9	0	9	100.00		
31-35Y (*N* = 6)	M	0	2	2	0.00	0.027*	50.00
	F	3	1	4	75.00		
36-40Y (*N* = 4)	M	0	1	1	0.00	-	50.00
	F	2	1	3	66.67		
40Y- (*N* = 4)	M	0	0	0	-	-	100.00
	F	4	0	4	100.00		
Total (*N* = 215)	M	58	37	95	61.05	-	79.53
	F	113	7	120	94.17		
*p* (Linear by linear association)	M				< 0.0001****		
	F				0.221		

Sep, number of samples with suture separation in each age subgroup and sex; Nonsep, number of samples with suture nonseparation in each age subgroup and sex; Subtotal, number of samples in each age subgroup and sex; Each sex, success rate of each sex in each age subgroup (%); Each age subgroup, success rate of each age subgroup (%); M, male; F, female

Bold values indicate the results that are statistically significant (*p < 0.05; **p < 0.01; ***p < 0.001; ****p < 0.0001)

The overall success rate of suture separation was 61.05% in male (mean age, 20.53 years; range, 8–38 years) and 94.17% in female (mean age, 19.58 years; range, 6–60 years), which resulted in a 79.53% success rate in all subjects (mean age, 20.00 years; range, 6–60 years). However, in subjects aged older than 15 years, the success rate decreased to 53.85% in male and 92.59% in female, which led to an overall success rate of 73.58%.

The linear-by-linear association between age subgroup and the success of suture separation in each sex showed a statistically significant association between the increase in age and suture nonseparation in male (*p* < 0.0001), but, not in female (*p* = 0.221).

Fisher’s exact test revealed an independent relationship between suture separation and sex in age subgroups younger than 20 years, while there was a strong association between both factors in age subgroups older than 21 years. In particular, in the age subgroup of 21–25 years, the odds of suture separation in female was 22.00 (95% confidence interval [CI]: 2.640–183.365) times that of male with suture separation.

### Suture separation ratio

Among the 215 included subjects, the suture separation ratio was evaluated in 171 (58 male and 113 female) subjects whose midpalatal suture was successfully separated. The suture separation ratio of each sex was normally distributed, as assessed by the Shapiro–Wilk test (male *p* = 0.289 and female *p* = 0.501).

In both sexes, the Jonckheere-Terpstra test indicated a statistically significant trend towards a low suture separation ratio in older age subgroups (*T*_JT_ = 242.00, *z* =  − 4.858, *p* < 0.0001 in male; *T*_JT_ = 1158.00, *z* =  − 6.047, *p* < 0.0001 in female) implying that despite the relatively similar condition of non-surgical maxillary expansion, the midpalatal suture expansion amount decreased with increasing chronological age.

**Fig. 5:**
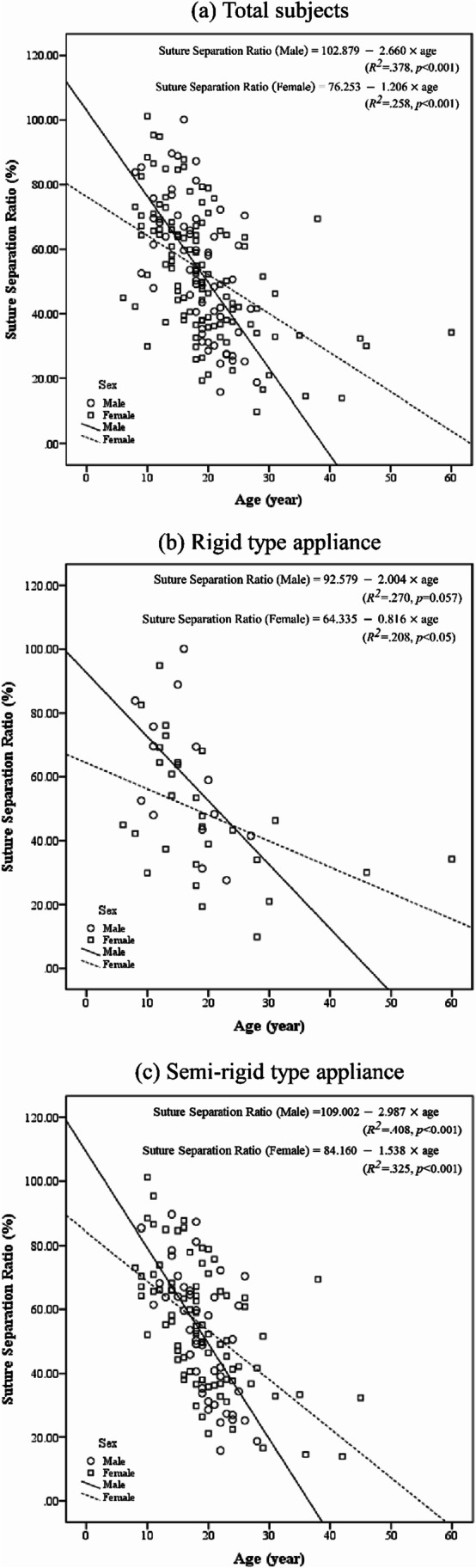
Analysis of linear regression between age (year) and the suture separation ratio (%) in male and female. **a** Total subjects. **b** Rigid type appliance. **c** Semi-rigid type appliance

Additionally, as numerical data, Pearson’s correlation revealed a statistically significant moderate degree of negative correlation between chronological age and the suture separation ratio (*r* =  − 0.615, *n* = 58, *p* < 0.001 in male; *r* =  − 0.515, *n* = 113, *p* < 0.001 in female). A linear regression model incorporating both rigid and semi-rigid type appliances established that age could statistically significantly predict the suture separation ratio in both sexes (Fig. 5a). In male, age accounted for 37.8% of the explained variability in the suture separation ratio, and the regression equation was as follows: predicted suture separation ratio = 102.879 − 2.660×age (*p* < 0.001). In female, age accounted for 25.8% of the explained variability in the suture separation ratio, and the regression equation was as follows: predicted suture separation ratio = 76.253 − 1.206×age (*p* < 0.001). With regard to the type of appliance, statistically significant regression models similar to the overall results of the subject population were drawn except for male with rigid type appliance (Fig. 5b, c). In both types of MARPE appliances, it was observed that the slope of the regression model in male was twice as greater as that in female, implying that the amount of suture separation is associated with chronological age and is considered somewhat irrelevant to the appliance.

**Fig. 6:**
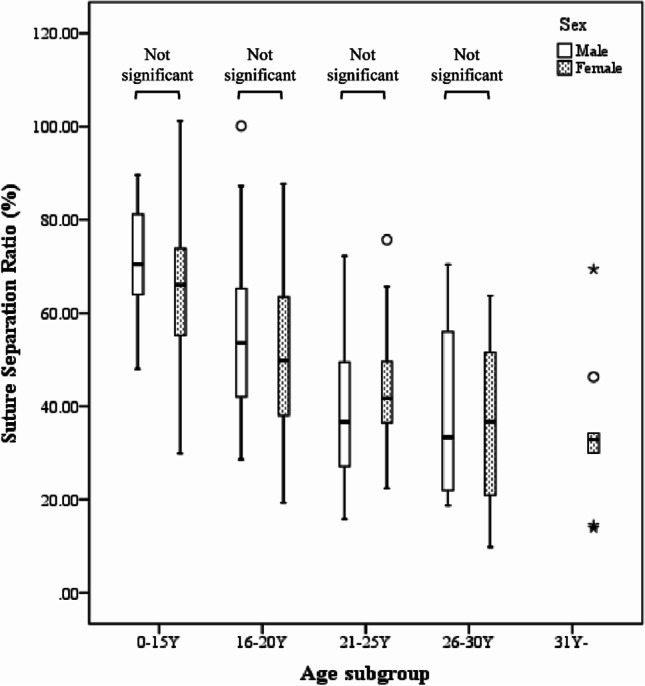
Changes in the suture separation ratio (%) according to age subgroup in male and female

In terms of sex, the Mann–Whitney *U* test indicated no statistically significant difference between the male and the female in all age subgroups (Fig. 6).

## Discussion

This study investigated the distribution and correlation of chronological age and sex with the success rate and suture separation ratio after the MARPE procedure. Our results revealed an apparent age-related difference between the male and the female in terms of the success rate of MARPE, and a significant correlation was found between chronological age and suture separation ratio.

As the use of the orthopedic appliances for nonsurgical maxillary expansion increases, efforts have been made to determine the presence of midpalatal suture maturation in order to predict the success of treatment [8, 11, 20]. However, several histological and micro-CT studies have indicated that chronological age and sex are not reliable parameters of midpalatal suture maturation [4, 13, 15, 18, 23, 24].

Histologically, the initiation of midpalatal suture obliteration differs from that of other sutures within the skull, which has been described as a continuous process that starts from the suture margins [23, 25]. Single bony islands of acellular and inconsistently calcified tissue, located within the suture or protruding as a small bone spicula from the suture margins, initiate the ossification process [23, 25]. Melsen [14] observed that midpalatal suture maturation starts from a broad and Y-shaped infantile stage and proceeds to the tortuous and interdigitated shape of the adolescent stage. Until now, however, it is not possible to determine whether there exists any obliteration area in each living individual since histological evaluation cannot be conducted by just reading radiographic images even with CT.

Furthermore, although the detailed mechanism and direction of midpalatal suture obliteration are known, the exact timing varies greatly among individuals [4, 13, 24]. According to the study by Angelieri et al. [24] using CBCT, neither chronological age nor sex and the maturational stages of the midpalatal suture had significant association. Korbmacher et al. [15] observed a significantly higher bone density in the sagittal dimension of the midpalatal suture in the middle-aged group than in the youngest and oldest age groups, whereas the obliteration index in the frontal plane exhibited substantial inter-individual variation and was independent of age. Such individual differences in suture obliteration may also occur under the influence of hormonal, genetic, and mechanical factors [23].

In addition to the histological characteristics of the midpalatal suture, mechanical resistance from the craniofacial complex and circumaxillary sutures occurs when the maxillary basal bone is subjected to attempted expansion. Therefore, predicting the success of MARPE by considering only the obliteration of the midpalatal suture may yield different results from the actual clinical findings. Lines [26] and Bell et al. [27] stated that the reason for the failure of nonsurgically assisted rapid maxillary expansion was increased rigidity of the facial skeleton in relation to the fusion of various combinations of zygomaticotemporal, zygomaticofrontal, and zygomaticomaxillary sutures.

With regard to the biomechanical effects of rapid maxillary expansion on the craniofacial structures, Işeri et al. [28], Gautam et al. [29], and Provatidis et al. [30] studied the finite element method. Işeri et al. [28] reported that high stress levels were observed in the canine and molar regions of the maxilla, lateral wall of the inferior nasal cavity, and zygomatic and nasal bones, with the highest stress concentration at the pterygoid plates of the sphenoid bone in the region close to the cranial base. Gautam et al. [29] demonstrated that both tensile and compressive stresses of variable magnitude were present along the same suture, particularly in the zygomaticomaxillary, zygomaticotemporal, and zygomaticofrontal sutures. Provatidis et al. [30] claimed that the zygomaticomaxillary suture influenced the response of the craniofacial complex to the expansion forces, and the sagittal suture at the level of the frontal portion of the midpalatal suture plays an important role in the degree and manner of maxillary separation.

The mechanical stress generated from rapid maxillary expansion may function as mechanical resistance, and the absolute magnitude of the induced stress depends greatly on bone elasticity and the patient’s age [29]. Even after active skeletal growth is completed, further maturation occurs in the circumaxillary suture sites [3]. Oberheim et al. [31] and Zioupos et al. [32] stated that the sutures of juvenile skulls absorb significantly higher bone strain than those of adults under equivalent orthopedic load, and the juvenile bone is generally less stiff than in adults, indicating that the same mechanical stress might cause different biological effects on immature and mature facial skeletons. Further, since bone mass distribution is distinguished between the male and the female due to sex-based differences such as periosteal expansion and endocortical apposition in bone maturation during puberty, the absolute values for parameters of bone strength are higher in male for a given cortical bone mass [33, 34]. Such changes in skeletal stiffness in both the craniofacial complex and circummaxillary sutures and the maturation of bone and/or sutures according to chronological age or sex may influence skeletal resistance during maxillary expansion, which can affect success rates or result in varying amounts of suture separation even after the success of suture separation.

In this study, the overall success rate of MARPE was 79.53% which was observed to be low compared with that of the meta-analysis by Kapetanović et al. [6] or previous studies on Koreans [9, 11]. Although the specific cause is uncertain, it is assumed to be the result of a relatively high proportion of older patients and male. In terms of sex-based difference, the success rate of MARPE was higher in female within every age subgroup at 92.59%, while that of male aged over 15 years decreased to 53.85%. In patients aged over 20 years, sex and the success of suture separation were statistically significantly associated. A statistically significant increase in the probability of suture nonseparation with increasing age was observed in male, and there were no successful cases in male aged over 30 years. This suggests that midpalatal suture maturation may not be related to chronological age or sex; however, from the perspective of skeletal stiffness or maturation in the craniofacial complex, there is a possibility that the midpalatal suture separation may be related to these factors.

Additionally, by evaluating the midpalatal suture expansion compared to the jackscrew expansion under the condition of a relatively similar mechanical stress applied to the maxilla, it was found that the difference in the suture separation ratio between sexes in the same age subgroup was statistically insignificant. However, the increase in chronological age was statistically significantly associated with a decrease in the suture separation ratio. According to the linear regression model incorporating both rigid and semi-rigid type appliance, the rate of change in the suture separation ratio as age increased was 2.21-fold greater in male (− 2.660) than in female (− 1.206). The analogous tendency was also observed in each type of MARPE. Therefore, it can be suggested that even if maxillary expansion by MARPE is successful, since resistance to expansion and the amount of skeletal expansion may change with chronological age or sex, treatment at a young age, particularly in male, would be advantageous not only for increasing the success rate of MARPE but also for achieving more skeletal expansion.

Meanwhile, although it was a minority, some of the subjects in this study were evaluated as nonseparation after the first 14 days of expansion; however, they were reevaluated as suture separation after the resumption of expansion following the 1 month resting phase. It is presumed that despite the failure of the midpalatal suture separation in the initial stage of expansion due to suture obliteration, the biological activity of the area increased during the resting period and suture separation was successful after the resumption of expansion. Alikhani et al. [35] demonstrated that the number and activity of osteoclasts, inflammatory cytokines, and chemokines significantly increased during the first month of expansion force application, and the bone density of the midpalatal suture was significantly decreased in the initial reaction of suture widening when transverse force was applied for the expansion of the midpalatal suture in rats. Taken together with the findings of this study, even if suture separation fails at the initial stage of expansion, it is assumed that if the subject undergoes a catabolic phase through the resting phase for approximately 1 month, firm suture obliteration may resolve to loose interdigitation through several biological reactions and the suture can be separated upon the resumption of expansion.

This study had a few limitations. First, the present study used a suture separation ratio calculated based on the values measured from a two-dimensional periapical radiograph to evaluate skeletal expansion. Although many recent studies have utilized CT images for three-dimensional analysis, there are ethical problems involved in acquiring CT images in a routine manner for all patients. Therefore, in the use of a periapical radiograph, the paralleling technique and consistent methodology were used to minimize the possibility of measurement errors due to image overlapping, distortion, and enlargement. Second, because of the retrospective and clinical nature of this study, the age distribution was somewhat unequal and the variety of transverse discrepancy in subjects resulted in a relatively heterogeneously controlled degree of expansion. To surmount the limitations of the two-dimensional analysis and to investigate the relevance between the success rate of MARPE and amount of suture separation with various clinical variables, such as anteroposterior and vertical skeletal patterns in addition to chronological age and sex, future case-controlled studies are warranted.

## Conclusion

Our findings suggest that the non-surgical maxillary expansion using MARPE may be a good approach for patients with transverse discrepancy; however, the success rate of MARPE and the suture expansion amount may depend on chronological age and sex. This study demonstrates that MARPE treatment in older patients, particularly in male, may result in a reduced likelihood of both success in suture separation and sufficient basal bone expansion followed by an unfavorable outcome of orthopedic expansion.

## Supplementary Information

Below is the link to the electronic supplementary material.

Supplementary file1 (XLSX 39 KB)
